# Impact of socioeconomic, demographic, maternal, infant and healthcare factors on early initiation of breastfeeding in Bangladesh: Evidence from Bangladesh demographic health survey (BDHS) 2022 data

**DOI:** 10.1371/journal.pone.0353716

**Published:** 2026-07-10

**Authors:** Nihar Ranjan Sikder, Bijoy Rajbongshi, Monir Hosen, Md. Mohibul Nasir Sopon, Afsana Mimi

**Affiliations:** Department of Statistics, University of Dhaka, Dhaka, Bangladesh; Flinders University, AUSTRALIA

## Abstract

**Background:**

Introducing an infant to the breast within an hour of birth is known as early initiation of breastfeeding (EIBF). Early initiation of breastfeeding (EIBF) was measured using BDHS 2022 data and defined as initiation of breastfeeding within one hour of birth, based on maternal self-report of the time elapsed between delivery and first breastfeeding. Using maternal self-reported timing of first breastfeeding from the Bangladesh Demographic and Health Survey (BDHS) 2022, this study examined factors associated with EIBF among mothers in Bangladesh.

**Methods:**

Data from 4,758 women who were fertile and had given birth were included in the study. To investigate the prevalence of EIBF and its association with different factors (socioeconomic, demographic, maternal, infant, and healthcare-related), descriptive analysis, and bivariate analysis using Pearson chi-square tests were carried out. Significant EIBF factors were found using binary logistic regression analysis.

**Results:**

Skin-to-skin contact within one hour of birth was significantly associated with higher odds of EIBF (OR = 1.62; 95% CI: 1.39–1.89). Cesarean delivery was associated with substantially lower odds of EIBF (OR = 0.40; 95% CI: 0.33–0.49). Compared with home deliveries, births in government facilities (OR = 0.58; 95% CI: 0.47–0.71) and private/NGO facilities (OR = 0.53; 95% CI: 0.42–0.66) were associated with reduced likelihood of EIBF. Significant regional variation in EIBF was observed across administrative divisions. Maternal age, education, and household wealth index were not consistently associated with EIBF after adjustment. Based on the univariate analysis, the prevalence of EIBF among mothers in Bangladesh is 63.3%. The 95% Confidence Interval for this prevalence is (61.9% – 64.7%).

**Conclusion:**

The significance of skin-to-skin contact for EIBF in Bangladesh is demonstrated in this study. The results indicate the necessity of focused initiatives to support EIBF, especially in medical settings and after cesarean deliveries. To understand the geographical differences in EIBF rates and create strategies to deal with them, more investigation is required. These discoveries can influence practice and policy to raise EIBF rates among Bangladesh’s varied demographics, improving the health of mothers and their offspring in the process.

## Introduction

The neonatal mortality rate has achieved a magical improvement over the last three decades [1]. Globally, it declined to 17 deaths per 1000 live births in 2022 which was 37 deaths per 1000 live births in 1990 [2]. In Bangladesh, the overall mortality rate declined to 23 deaths per 1000 births in 2022 which was 100 deaths per 1000 births in 1990 [3]. Regardless the rate is high compared to the SDG goal of 12 deaths per 1000 live births [4].

Infant breastfeeding should begin as soon as possible to minimize the risk of newborn death rate [5]. EIBF stands for early initiation of breastfeeding, which means feeding the baby within one hour after delivery [6]. The production of colostrum, a nutrient-rich “first milk” that boosts the newborn’s immune system, lowers the risk of infection, promotes mother-child bonding, stabilizes the child’s blood sugar levels, and controls body temperature, is stimulated by early breastfeeding [7]. Additionally, by encouraging uterine contractions, early breastfeeding helps mothers undergo less postpartum hemorrhage [8]. Most likely infections are more prevalent in newborns whose mothers do not start breastfeeding after the first hour after birth (33%) [9], have a 50% higher chance of respiratory problems throughout the first six months of life as well as a greater likelihood of death compared with those whose mothers begin breastfeeding within 1 hour of birth [10,11].

Around the world, the majority of newborns (52%) are not receiving early breastfeeding, even though it has far too many advantages [12]. There are many reasons for late breastfeeding initiation. Breastfeeding can be delayed by a mother’s lack of knowledge and understanding of the significance of breastfeeding a child at a young age, as well as by misconceptions and incomplete information [13]. The cultural norms and customs followed in different cultures around the world. For instance, some cultures are known for pre-lacteal feeding, which involves giving infants something other than breast milk before they are breastfed [14]. Immediate postnatal practices, such as separating the mother and child, can also be a barrier in many hospitals since they delay the start of breastfeeding and provide insufficient training and support for medical staff [7].

In Bangladesh, a number of reasons prevent children from beginning breastfeeding at an early age such as maternal age, education and occupation, socioeconomic status, cultural factors, places of residence, antenatal care (ANC) and type of delivery [15–18]. Particularly in rural areas, where there is typically a lack of proper healthcare infrastructure, greater obstacles are encountered [19]. In certain regions of the country, there are still many people who adhere to traditional beliefs and customs, such as discarding away colostrum and offering babies honey or sugar water before breastfeeding [20]. Even with developments, there are still insufficient qualified medical professionals to carry out the early breastfeeding program. Some hospitals may not adhere strictly to the requirements set forth by the Baby-friendly Hospital Initiative (BFHI) [21]. Mothers who had cesarean sections rarely breastfeed their babies at first. Additionally, after giving birth to a child, women with higher body mass index (BMI) delay breastfeeding [22].

Though early breastfeeding significantly reduces neonatal mortality and has long-term health benefits for both mother and child, only 42.6% [5] around the world and only 51.4% [15] mothers in Bangladesh initiate breastfeeding within one hour of birth. The skin-to-skin touch of child with their mothers has a positive impact on early initiation of breastfeeding [23,24]. The purpose of this study is to re-investigate the impact of skin-to-skin touch of child with their mothers on early initiation of breastfeeding in Bangladesh.

In all the past research, researchers discovered socioeconomic inequalities in early breastfeeding initiation in Bangladesh [25]. In another research study named gender inequality in early Initiation of Breastfeeding, it is found that the study review highlights gender disparities in early breastfeeding [26]. This research shows that initially, female children were less likely to be nursed in the first hour compared to males, but this gap decreased over time. The article “Skin-to-skin contact and early initiation of breastfeeding in Bangladesh: a cross-sectional study using MICS6, Bangladesh (2019) data” explores the low rates of these practices in Bangladesh and identifies the factors influencing them based on the 2019 MICS6 data [27].

While this study uses cross-sectional BDHS 2022 data, it effectively investigates the decline in EIBF by identifying that modern barriers like high cesarean rates (45.7%) and low skin-to-skin contact (17-25.7%) are currently the strongest predictors of late initiation. The research fills a critical gap by highlighting that institutional births in Bangladesh are now significantly less likely to practice EIBF than home deliveries, providing a necessary evidence base for urgent policy intervention. We explored in this paper which factors are associated with these changes and how the EIBF rate can be increased in Bangladesh. In this paper, we aim to investigate the associations of various socioeconomic, demographic, maternal, infant, and maternal factors on early initiation of breastfeeding in Bangladesh using Bangladesh Demographic Health Survey (BDHS) data 2022.

## Methods and materials

### Data

This study performed a secondary analysis of data from the 2022 Bangladesh Demographic and Health Survey (BDHS) [28], focusing on 4,758 women of reproductive age who had given birth for early initiation of breastfeeding practices. Data from the 2022 BDHS [28], a nationally representative survey that includes every person residing in non-institutional housing in the People’s Republic of Bangladesh, was used in the study. The survey sampling frame was based on the 2011 Population and Housing Census of Bangladesh, provided by the Bangladesh Bureau of Statistics (BBS). The primary sampling unit (PSU) was an enumeration area (EA) with approximately 120 households on average. There are eight administrative divisions in Bangladesh: Barisal, Rangpur, Rajshahi, Chattogram, Dhaka, Khulna, Mymensingh, and Sylhet. Each division is further subdivided into zilas and each zila into upazilas, facilitating the categorization of the country into urban and rural areas. The survey was based on a two-stage stratified sampling method. In the first stage, 675 EAs (237 urban and 438 rural) were selected with a probability proportional to their EA size. This sample was drawn by BBS, according to specifications provided by the Inter-City Fund (ICF), which included guidelines on cluster allocation and sample selection. A comprehensive household listing operation was conducted in all selected EAs to create a sampling frame for the second-stage household selection. In the second stage, a systematic sample of approximately 45 households per EA was chosen to ensure statistically reliable estimates of key demographic and health variables for the entire country, as well as for urban and rural areas separately, and for each of the eight divisions. This sampling design resulted in the selection of 30,330 residential households, with completed interviews anticipated from around 30,358 ever-married women aged 15–49 years.

### Figure: Respondent Selection

#### Statistical analysis.

The proportion of women who initiated EIBF is reported as a percentage. Simple logistic regressions were performed between each explanatory variable and the outcome variable among mothers. Odds ratios of significantly associated explanatory variables with 95% confidence intervals (CI) are reported. A p-value <0.05 was considered statistically significant for all analyses performed. Statistical analysis was performed using SPSS version 27. We used BDHS 2022 data.

### Ethical consideration

The 2022 Bangladesh Demographic and Health Survey (2022 BDHS) [28] was conducted under the authority of the National Institute of Population Research and Training (NIPORT), Medical Education and Family Welfare Division, Ministry of Health and Family Welfare (MOHFW). The survey was implemented by Mitra and Associates, a private research agency, from June 2022 to December 2022. Participants provided their consent for the study by signing or, if they were illiterate, by providing a thumbprint. No personal identifiers were included in the data before it was made publicly available.

### Variables

The World Health Organization (WHO) and UNICEF recommended that mothers should start early initiation of breastfeeding within 1 hour of birth for infants [29]. According to this, the outcome variable for this study is classified into two categories: whether a mother breastfeeds her child within 1 hour of birth or not.

To take into account the association of covariates with early initiation of breastfeeding, socioeconomic, demographic, maternal, infant, and healthcare-related variables are considered by reviewing previous literature. Within socioeconomic covariates, the mother’s education [22,27,30–33] and wealth index [26,34,35] are taken into consideration. Mother’s education was categorized into 4 groups (no education, primary education, secondary education, and higher education). Wealth index was grouped as: poor, middle, and rich. Place of residence (urban and rural) [22,25,26,30,32,35–37], division (Barisal, Chittagong, Dhaka, Khulna, Mymensingh, Rajshahi, Rangpur, Sylhet) [25,26,30,37] are considered as demographic variables for this study. In the maternal covariates, this study considered the age of the mothers at last birth [26,34], the birth order [25,38] and healthcare decisions [22,38]. The mother’s age at last birth was categorized into three groups: (age below 20, age between 21–30, age greater than 30). Birth order grouped as: (1^st^, 2^nd^, 3^rd^ and above). Decisions on mother’s healthcare was divided into two sub-groups: mother (mother alone, mother jointly with partner/other person) and others (husband alone, someone else, other people). Further, the study considered the sex of child [22,25,26,30–33,37,39] and skin-to-skin touch (put on mother’s chest) [22,36] as infant factors. Sex of child was categorized into male and female, skin-to-skin touch (child put on mother’s chest and bare skin) was categorized into two groups as whether put on chest or not. Furthermore, in this study, place of delivery [25,30,33] and mode of delivery [22,25,26,33,39] were considered as healthcare factors. Place of delivery was labeled into three categories: respondent`s home, government facilities, and NGO/ private/ others. Mode of delivery was classified into two categories: delivery by cesarean or not.

## Results

In this study, since all the variables are considered as categorical variables, percentage distribution for each category of each variable is calculated. Descriptive statistics for the selected dependent variable and covariates are reported in Table 1.

**Table 1:** Descriptive analysis for the selected dependent variable and covariates.

Variable	Category	Frequency	Valid Percentage (%)
**Child breastfeeding initiation within one hour**	yes	3011	63.3
	No	1747	36.7
**Age at last birth**	Below 20	1088	22.9
	20-30	2757	57.9
	Above 30	913	19.2
**Place of residence**	Urban	1564	32.9
	Rural	3194	67.1
**Division**	Barisal	513	10.8
	Chittagong	816	17.2
	Dhaka	705	14.8
	Khulna	540	11.3
	Mymensingh	593	12.5
	Rajshahi	486	10.2
	Rangpur	557	11.7
	Sylhet	548	11.5
**Highest education level**	No Education	255	5.4
	Primary	1094	23.0
	Secondary	2497	52.5
	Higher	912	19.2
**Birth order number**	1	1772	37.2
	2	1663	35.0
	3 or above	1323	27.8
**Wealth**	Poor	1946	40.9
	Middle	947	19.9
	Rich	1865	39.2
**Sex of child**	Male	2449	51.5
	Female	2309	48.5
**Place of delivery**	Respondents` home	1680	35.3
	Government facilities	811	17.0
	NGO/Private/Others	2267	47.6
**Delivery by cesarean section**	Yes	2576	45.7
	No	2172	54.3
	Missing/Unknown	10	0.2
**Skin to skin touch**	Yes	1222	25.7
	No	3536	74.3
**Who decides on healthcare**	Respondents	3387	71.3
	Others	1332	25.7
	Missing/Unknown	39	0.8

From the univariate analysis, 63.3% of mothers initiated breastfeeding within one hour of childbirth, while 36.7% initiated it after one hour. Regarding the age at last birth category, 22.9% of respondents are below 20 years, 57.9% are in the 20–30 years age category, and 19.2% are above 30 years. In terms of place of residence, 32.9% of respondents live in urban areas, while 67.1% live in rural areas. In the division category, 10.8% of respondents are from Barisal, 17.2% from Chittagong, 14.8% from Dhaka, 11.3% from Khulna, 12.5% from Mymensingh, 10.2% from Rajshahi, 11.7% from Rangpur, and 11.5% from Sylhet. Regarding education level, 5.4% of respondents have no education, 23% have completed primary education, 52.5% have completed secondary education, and 19.2% have completed higher education. In terms of birth order, 37.2% of respondents gave birth to their first child, 35% gave birth to their second child, and 27.8% gave birth to their third or subsequent child. In the wealth index category, 40.9% of respondents belong to the poor class, 19.9% to the middle class, and 39.2% to the rich class. In terms of the sex of the child, 51.5% are male, and 48.5% are female. Additionally, 35.3% of respondents chose their home for delivery, 17.4% opted for government facilities, and 47.6% preferred NGO/private/other places for delivery. Around 45.7% of respondents had a cesarean section for delivery, while 54.3% did not. After the birth of the child, only 25.7% experienced skin-to-skin contact with their mothers, while 74.5% were deprived of that experience. In 71.3% of cases, respondents made their own healthcare decisions, while in 28.2% of cases, others made the healthcare decisions.

For the variables “Delivery by cesarean section” and “who decides on healthcare”, a small number of observations had missing values (32 and 9, respectively). Given that these represented only a very small proportion of the total sample, we categorized these missing responses as ‘Unknown’ and retained them in the analysis. This approach allowed us to preserve the full sample size while acknowledging the presence of missing data in the descriptive tables and regression models.

To explore the unadjusted association between early initiation of breastfeeding and certain covariates, a bivariate analysis was conducted using the Pearson chi-square test, and the results are displayed in Table 2.

**Table 2:** Percentage distribution of child breastfeeding initiation among different categories of selected covariates along with p-value.

Variable	Category	Child breastfeeding initiation within one hour		p-value
**Yes**	**No**
**Age at last birth**	Below 20	61.7	38.3	0.018
	20-30	64.9	35.5	
	More than 30	60.2	39.8	
**Place of Residence**	Urban	59.3	40.7	<0.001
	Rural	65.2	34.8	
**Division**	Barisal	60.2	39.8	<0.001
	Chittagong	64.7	35.3	
	Dhaka	57.0	43.0	
	Khulna	53.7	46.3	
	Mymensingh	63.7	36.3	
	Rajshahi	60.7	39.3	
	Rangpur	70.7	29.3	
	Sylhet	75.7	24.3	
**Highest Education Level**	No Education	63.9	36.1	<0.001
	Primary	68.4	31.6	
	Secondary	62.6	37.4	
	Higher	58.9	41.1	
**Wealth**	Poor	68.9	31.1	<0.001
	Middle	63.3	36.7	
	Rich	57.5	42.5	
**Birth Order Number**	1	59.0	41.0	<0.001
	2	63.5	36.5	
	3 or above	68.7	31.3	
**Sex of Child**	Male	62.9	37.1	0.555
	Female	63.7	36.3	
**Place of Delivery**	Respondent`s Home	80.2	19.8	<0.001
	Government Facilities	63.1	36.9	
	NGO/Private/Others	50.8	49.2	
**Delivery by Cesarean**	Yes	47.1	52.9	<0.001
	No	67.9	23.1	
**Skin to Skin Touch**	Yes	72.7	27.3	<0.001
	No	60.0	40.0	
**Who Decides on Healthcare**	Respondent	62.6	37.4	0.109
	Others	65.1	34.9	

From Table 2, it is observed that all covariates were significantly associated with early initiation of breastfeeding except for the sex of the child and who decides on healthcare. There was a significant association between the mother’s age at last birth and early breastfeeding initiation (p-value = 0.018). Mothers aged 20–30 showed the highest rate of early initiation (64.9%), while those over 30 years of age had the lowest rate (60.2%). In contrast, mothers under 20 years old had an early initiation rate of 61.7%, this association was significant. Additionally, the place of residence had a significant association with breastfeeding initiation (p-value < 0.001); notably, 65.2% of rural mothers began breastfeeding within one hour, whereas only 59.3% of urban mothers did so. The geographical region was also statistically associated with early initiation of breastfeeding rates at a 5% level of significance (p-value < 0.001), where mothers from Sylhet and Rangpur had the highest rates at 75.7% and 70.7%, respectively. Conversely, mothers from Khulna and Dhaka exhibited lower rates, with 53.7% and 57.0%, respectively. Moreover, maternal education was another significant factor (p-value < 0.001); mothers with only primary education had a higher tendency (68.4%) to initiate breastfeeding within one hour, while those with higher education had a lower tendency (58.9%). However, among mothers with no education, the rate was 63.9%. Furthermore, economic status also had a significant impact on early breastfeeding initiation at a 5% level of significance (p-value < 0.001). Mothers from poor households had a higher tendency (68.9%) to initiate breastfeeding within one hour, while mothers from wealthy families showed a lower rate (57.5%). However, mothers from middle-income families had an initiation rate of 63.3%. Additionally, mothers with a child whose birth order was third or more showed the highest initiation rate (68.7%); however, it was 63.5% for second-order births and 59.0% for first-order births, with a p-value < 0.001 indicating significant associations. On the other hand, 80.2% of respondents initiated breastfeeding within one hour if they delivered at home, 63.1% if they delivered in government facilities, and only 50.8% if they delivered in NGO/private/other facilities. This association was statistically significant at a 5% level of significance, with a p-value < 0.001. Another significant factor was delivery by cesarean (p-value < 0.001), showing that if respondents delivered by cesarean section, only 47.1% initiated breastfeeding within one hour, whereas 67.9% of those who delivered vaginally initiated breastfeeding within one hour. Lastly, skin-to-skin contact showed a strong association with breastfeeding initiation (p-value < 0.001). Among mothers who had skin-to-skin contact, 72.7% initiated breastfeeding within one hour, while only 60% of those without skin-to-skin contact did so.

The associations of socioeconomic, demographic, maternal, infant, and healthcare factors with early breastfeeding initiation are determined using a binary logistic regression model due to the binary outcome variable. To achieve this goal, the binary logistic model has been run to address the control effects of each category of covariates. The results of the binary logistic regression model display the model’s intercept, standard error, p-value, odds ratio (OR), and 95% confidence interval (CI) of OR in Table 3.

**Table 3:** Estimated regression coefficient, standard error (S.E), p-value, odds ratio (OR), and 95% confidence interval (CI) of OR, obtained from binary logistic regression model for child breastfeeding initiation within one hour of birth.

Variables	Category	β	S.E.	P-value	Odds	95% C.I. of Odds	
						**Lower**	**Upper**
**Intercept**		0.687	0.189	<0.001 ***	1.988		
**Age at last birth**	Below 20 (Ref)						
	20-30	0.012	0.096	0.902	1.012	0.839	1.220
	Above 30	−0.280	0.130	.032 **	0.756	0.586	0.976
**Place of Residence**	Urban Rural (Ref)	−0.027	0.070	0.695	0.973	0.848	1.116
**Division**	Barisal	−0.110	0.127	0.388	0.896	0.699	1.149
	Chattogram	0.044	0.113	0.700	1.044	0.837	1.303
	Dhaka (Ref)						
	Khulna	0.016	0.123	0.897	1.016	0.799	1.292
	Mymensingh	0.085	0.123	0.491	1.089	0.855	1.386
	Rajshahi	0.167	0.128	0.192	1.182	0.920	1.518
	Rangpur	0.503	0.128	<0.001 ***	1.654	1.287	2.127
	Sylhet	0.479	0.135	<0.001 ***	1.615	1.239	2.105
**Education Level**	No Education (Ref) Primary	0.359	0.156	0.022 **	1.431	1.054	1.944
	Secondary	0.425	0.151	0.005 ***	1.529	1.138	2.055
	Higher	0.656	0.166	<0.001 ***	1.928	1.391	2.670
**Wealth Index**	Poor (Ref)						
	Middle	0.012	0.081	0.886	1.012	0.863	1.185
	Rich	0.012	0.081	0.886	1.012	0.863	1.185
**Birth Order Number**	1 (Ref)						
	2	0.236	0.087	0.007 ***	1.266	1.068	1.501
	3 or more	0.319	0.111	0.004 ***	1.376	1.108	1.709
**Gender**	Male Female (Ref)	−0.005	0.064	0.937	0.995	0.877	1.129
**Place of Delivery**	Respondent Home (Ref)						
	Govt. Facilities	−0.549	0.108	<0.001 ***	0.577	0.467	0.713
	NGO/Private/Others	−0.639	0.112	<0.001 ***	0.528	0.423	0.657
**Delivery by Cesarean**	Yes No (Ref)	−0.907	0.096	<0.001 ***	0.404	0.334	0.487
**Skin to Skin Touch**	Yes No (Ref)	0.482	0.078	<0.001 ***	1.619	1.389	1.887
**Who Decides on Healthcare**	Respondent Others (Ref)	−0.077	0.073	0.291	0.926	0.803	1.068

Note: Ref = reference; S.E. = Standard Error.

*** = significant at 1% level of confidence, ** = significant at 5% level of significance.

The findings from Table 3 highlight that mothers aged above 30 were less likely to initiate early breastfeeding compared to mothers aged below 20. This is reflected in the odds ratio of 0.756 (p-value = 0.032), indicating approximately 24.4% lower odds of early initiation of breastfeeding compared to mothers aged below 20. However, mothers aged between 20–30 years showed no significant associations with early initiation of breastfeeding. On the other hand, mothers from the Rangpur and Sylhet divisions were more likely to initiate early breastfeeding in contrast with mothers from the Dhaka division, as observed in odds ratios of 1.654 (P-value<0.001) and 1.615 (P-value<0.001), respectively, representing 65.4% and 61.5% higher odds of early initiation of breastfeeding relative to the Dhaka division. Mothers belonging to the other divisions (Barishal, Chattogram, Khulna, Mymensingh, and Rajshahi) did not show significant associations with early initiation of breastfeeding. Moreover, mothers who received primary, secondary, and higher education significantly influenced early initiation of breastfeeding, with p-values of 0.022, 0.005, and <0.001, respectively. Specifically, mothers with primary, secondary, and higher education have odds ratios of 1.431, 1.529, and 1.928, respectively, indicating 43.1%, 52.9%, and 92.8% higher odds of early breastfeeding initiation compared to mothers with no education. Furthermore, mothers having a child whose birth order was second show an odds ratio of 1.266, representing 26.6% higher odds of early breastfeeding initiation as opposed to those mothers having a first-born child. Similarly, mothers with a child whose birth order was three or more have an odds ratio of 1.376, reflecting 37.6% higher odds compared to mothers whose child’s birth order was first. Thus, these associations are statistically significant at a 5% level of significance with p-values of 0.007 and 0.004, respectively. Additionally, mothers who delivered at government facilities or NGO/private/other facilities have lower odds (OR=0.577, OR=0.528) of initiating early breastfeeding compared with those who delivered at home, which shows a significant association (p-value<0.001, p-value<0.001. Finally, the odds ratio of mothers who delivered via cesarean section was 0.404, indicating 59.6% lower odds of early breastfeeding initiation compared to those who delivered vaginally. This association is highly significant (p-value < 0.001). Additionally, mothers who placed their child on their chest after birth, known as skin-to-skin touch, have an odds ratio of 1.619, representing 61.9% higher odds of early breastfeeding initiation compared to those who did not. This association is also highly significant (p-value < 0.001). Variables were selected for inclusion in the logistic regression model based on theoretical relevance and prior literature, rather than statistical significance in bivariate analysis alone. Accordingly, child’s sex and decision-making authority for healthcare were retained as potential confounders.

## Discussion

The early initiation of breastfeeding is a low-cost, effective intervention with far-reaching health and socio-economic benefits for families in Bangladesh. It enhances infant survival, supports maternal health, reduces healthcare costs, and aligns with national health goals, making it a critical focus for improving public health outcomes in the country [40].Therefore, it is crucial to determine the factors that promote early breastfeeding initiation and those that lead to late breastfeeding initiation.

In this study, it is found that mothers aged above 30 had a negative impact on early initiation of breastfeeding. The rate of breastfeeding decreases when the mother’s age is above 30. Within divisions, there were variations in the prevalence of EIBF as well. From Table 3, it is found that mothers from Rangpur and Sylhet divisions had a positive impact on initiating early initiation of breastfeeding, indicating that they are conscious about their newborns’ health and mothers’ health. Variations in the frequency of early initiation of breastfeeding among various geographic areas may be explained by differences in the availability of healthcare facilities and information linked to healthcare [15,34]. In this study, there is also a greater prevalence of early initiation of breastfeeding beginning among those with higher education levels. Similarly, a number of previous studies have shown that mothers who received higher education were statistically associated with a higher likelihood of starting breastfeeding early [41–44]. These findings represent that mothers who are highly educated are more conscious about the necessity of early initiation of breastfeeding for their infants as well as for their health. Also, this study observed that early initiation of breastfeeding increases as birth order increases which is 3rd or above. This finding complies with some research conducted in Namibia and Ethiopia [45,46]. Children who were born first were more at risk of not starting breastfeeding at early stages. Changes in knowledge, awareness, beliefs, and behaviors surrounding nursing techniques may have occurred in mothers with multiple children [45,47]. Higher odds of delayed breastfeeding beginning for firstborn children may result from mental discomfort, ignorance, lack of support from family or medical professionals, and lack of preparedness, among other factors [48,49]. In addition, there is a higher chance of problems during delivery particularly in cases of first birth [50]. Most of those cases involved cesarean birth, which has been established to be an obstacle to starting breastfeeding on time [51]. A baby delivered via cesarean section spends some time apart from the mother. It has been noted that first-time mothers encounter more difficulties when it comes to breastfeeding [52,53]. Even though they intended to breastfeed, some of them acknowledged insecurity [49]. Mothers who gave birth in a hospital or clinic were negatively influenced to practice EIBF compared to mothers who delivered at home. A similar finding was found in Ahmmed and Manik’s investigation [34]. This result was in contrast to previous studies conducted in Tanzania, Nepal, and Ethiopia, which did not discover any significant correlation with the delivery location [15,45,54,55]. Also, this finding goes contrary to an Indian study that discovered mothers who gave birth at home were less likely to perform EIBF than mothers who gave birth in hospitals [56]. Also, in this study, it is found that mothers who were delivered at NGO/Private/Others were also negatively associated with EIBF compared to mothers who delivered at home. The prevalence of EIBF was considerably reduced among mothers who delivered via cesarean section compared with those who delivered regularly. This result supported findings from Tanzania, Ethiopia, and Bangladesh [46,47,57]. This may be explained by the fact that women who give birth via cesarean section are separated from their newborns for the duration of the surgical process. It is more common for mothers giving birth in an institution to do so by surgery or cesarean section. It is challenging for mothers to awaken from anesthesia within an hour after giving birth in order to nurse their children as the majority of surgeries are conducted under general anesthesia [34]. Therefore, it is advised to provide clear guidelines on EIBF following a cesarean section and to raise awareness of the significance of EIBF among all women and healthcare professionals. In this study, it is discovered that early initiation of breastfeeding within 1 hour of birth is strongly positively associated with skin-to-skin touch immediately after childbirth in Bangladesh. The findings of the present study suggest that infants who had skin-to-skin touch after birth plays a significant role in early initiation of breastfeeding. Through the production of the hormones “oxytocin,” which promotes bonding between mother and newborn, and “prolactin,” which encourages lactation, skin-to-skin contact with the mother helps start the process of breastfeeding earlier [58]. Furthermore, This study revealed that there is no significant association with the sex of child and early initiation of breastfeeding within 1 hour, which reflects that gender equality exists. By improving early initiation of breastfeeding, newborn morbidity and mortality would likely decrease and contribute to achieving the Sustainable Development Goals (SDG) 2030 for reducing child mortality [39]. The apparent difference between maternal age and parity reflects their independent adjusted effects in the multivariable model. While age may be associated with delivery-related constraints, higher parity likely promotes EIBF through prior breastfeeding experience. The lower odds of early initiation of breastfeeding (EIBF) among mothers who delivered in government or private health facilities compared with those who delivered at home may reflect facility-level and delivery-related factors rather than reduced awareness of breastfeeding practices. Facility births are more likely to involve medical interventions such as caesarean section, delayed mother–infant contact, routine newborn procedures, or separation of the mother and infant, all of which can delay breastfeeding initiation. In addition, inconsistent implementation of breastfeeding-friendly practices, including immediate skin-to-skin contact and post-delivery counseling, may contribute to delayed EIBF in some facilities. These findings underscore the need for further investigation into institutional barriers within both government and private health facilities to ensure adherence to recommended early breastfeeding practices. At present, EIBF percentages declined according to BDHS 2022 data [28] compared to BDHS 2017−18 data [59]. The percentage rate was 79.2% according to BDHS 2017−18 data [59], however, the percentage is 63.3% according to BDHS 2022 [28].

This study shows that we need to change how hospitals and clinics handle the first hour of a baby’s life to save more newborns in Bangladesh. Since placing a baby directly on the mother’s chest (skin-to-skin contact) is the biggest booster for early breastfeeding, this should be a standard rule for every birth. We also need to fix the “hospital barrier”, it is concerning that mothers giving birth at home are often more successful at early breastfeeding than those in modern facilities. This means hospitals, especially for C-sections, must stop separating moms and babies and provide better hands-on help. Finally, we should give extra support to first-time mothers and keep investing in girls’ education, as more educated women are nearly twice as likely to start breastfeeding right away.

## Conclusion

Using the BDHS Data from 2022, we investigate the variables related to early breastfeeding initiation among mothers in Bangladesh in this study. Here, we discovered that a number of factors affect the beginning of early breastfeeding, such as the Division, Education, Birth order number, place of delivery, Delivery by Cesarean and Skin-to-skin contact.

This study has provided useful information for improvement by identifying important factors impacting the early initiation of breastfeeding. Mothers from Rangpur and Sylhet divisions have exhibited positive impact of early breastfeeding compared to those in Dhaka, suggesting the need for targeted interventions in regions with lower rates. The importance of encouraging female education is highlighted by the fact that maternal education is a powerful predictor, with women having primary, secondary, and higher education increased probabilities of initiating breastfeeding early.

Another important factor is birth order, mothers with second and higher-order children were plausible to initiate breastfeeding early, suggesting that first time mothers should get special attention from healthcare experts. Opposed to home deliveries, institutional deliveries especially those in public and private facilities were linked to reduced probabilities of early breastfeeding, highlighting the need for improved breastfeeding assistance in healthcare settings. Cesarean deliveries further reduced early initiation of breastfeeding rates, underscoring the necessity for structured post-operative breastfeeding support.

Lastly, early breastfeeding initiation was greatly aided by skin-to-skin touch, supporting its broad inclusion in delivery methods [60]. By addressing these factors through targeted interventions, health education, and systemic changes, early breastfeeding practices can be significantly improved, leading to better maternal and child health outcomes.

According to the BDHS report 2014, 25% of newborns were put on mothers chest immediately after birth [61]. However, the rate declined according to the BDHS report 2017−18, it was only 14% skin-to-skin contact [59]. But the rate slightly increased according to the BDHS report 2022, which was only 17% skin-to-skin contact [28]. So, proper steps should be taken to increase the rate of skin to skin contact along with early initiation of breastfeeding.

In summary, our research reveals significant variables that impact the early initiation of breastfeeding in Bangladesh. By focusing on particular methods, including encouraging skin-to-skin contact and normal deliveries, early initiation of breastfeeding rates can be increased. Also, steps should be taken to enhance early initiation of breastfeeding rate for the delivery cases which are taken place in healthcare facilities. Enhancing breastfeeding practices can lead to better health outcomes of mother and child. Policy efforts should prioritize ensuring immediate skin-to-skin contact and breastfeeding support after both vaginal and cesarean deliveries, particularly in health facilities. Strengthening breastfeeding counseling for first-time mothers and improving adherence to Baby-Friendly Hospital Initiative guidelines can help increase EIBF rates in Bangladesh. This study is based on cross-sectional BDHS data; therefore, causal relationships between identified factors and early initiation of breastfeeding cannot be established. In addition, the timing of breastfeeding initiation was self-reported by mothers, which may be subject to recall bias.
